# Transmesosigmoid hernia in a 30-year-old patient: A rare case report and surgical management

**DOI:** 10.1016/j.ijscr.2025.111842

**Published:** 2025-08-18

**Authors:** Ibrahim Fathallah, Ahmed Al-Talep, Abd Alrhman Alajrd, Abd Al-Hadi Al-Souqi, Saleh Saleh

**Affiliations:** a Faculty of Medicine, Homs University, Homs, Syria; b Faculty of Medicine, Damascus University, Damascus, Syria

**Keywords:** Internal hernia, Transmesosigmoid hernia, Sigmoid colon, Small bowel obstruction, Case report

## Abstract

**Introduction and clinical importance:**

Internal hernias (IHs) are uncommon, representing under 1 % of all cases, and are hard to diagnose early, often leading to discovery during surgery.

**Case presentation:**

A 30-year-old patient with no prior surgeries presented with sudden severe abdominal pain and vomiting. Examination showed generalized defence and hyperactive bowel sounds. Imaging suggested bowel obstruction. Emergency surgery revealed a large internal hernia through the right leaflet of an elongated sigmoid mesocolon containing the entire small bowel. The bowel was viable and reduced, followed by sigmoid resection and temporary colostomy. The patient recovered well with no major complications and was discharged on day eight.

**Clinical discussion:**

Internal abdominal hernias are a rare cause of small bowel obstruction (SBO), with transmesosigmoid hernias (TMSHs) being one of the less common subtypes. These hernias occur due to defects in the sigmoid mesocolon and can present with acute abdominal pain and signs of bowel obstruction. Although both congenital and acquired factors have been suggested, the exact cause remains unclear. Prompt diagnosis is crucial to avoid serious complications such as strangulation, gangrene, and perforation. Multidetector CT scans have greatly improved preoperative diagnosis by providing detailed images of internal structures. Surgical repair, either laparoscopic or open, is essential for treatment, with laparoscopy offering benefits in terms of recovery and complications.

**Conclusion:**

This report emphasizes the rarity of TMSHs and difficult to diagnose due to nonspecific symptoms. Moreover, highlights the value of early diagnosis and timely surgical intervention to prevent complications.

## Highlights

• Internal hernias are rare but important causes of small bowel obstruction, representing less than 1 % of cases.
• Transmesosigmoid hernias are an uncommon type involving a full-thickness defect of the sigmoid mesocolon.
• Surgical exploration remains essential for diagnosis and treatment, with options including open or laparoscopic repair.
• Timely recovery after surgery and close medical follow-up play a key role in ensuring a positive outcome for the patient.

## 1 Introduction

IHs are rare but significant causes of SBO, accounting for less than 1 % of all cases [1]. They are characterized by the protrusion of a visceral organ, typically a segment of the small intestine, through a peritoneal or mesenteric defect without an external sac. Among the various types of IHs, sigmoid mesocolon hernias are among the rarest, especially TMSHs, which involve a full-thickness defect through both peritoneal layers of the sigmoid mesocolon [2,3]. Clinical presentation of IHs is often nonspecific and may mimic other causes of acute abdomen. Patients frequently present with crampy abdominal pain, vomiting, and signs of bowel obstruction. Laboratory findings may show leukocytosis, but vital signs can be initially stable. The diagnosis is often challenging and delayed due to the lack of pathognomonic signs, and many cases are diagnosed only at the time of surgery [4,5].

In recent years, contrast-enhanced computed tomography (CT) has improved the preoperative diagnostic accuracy of internal hernias, with characteristic findings such as clustered bowel loops, mesenteric vessel crowding, and closed-loop obstructions. However, CT findings can still be inconclusive in urgent presentations [6]. This report describes a case of TMSH in a 30-year-old man, emphasizing the diagnostic and treatment challenges of this unique presentation. This case report has been reported in line with the 2025 SCARE criteria to ensure transparency and high reporting quality [7].

## 2 Case presentation

**Fig. 1:**
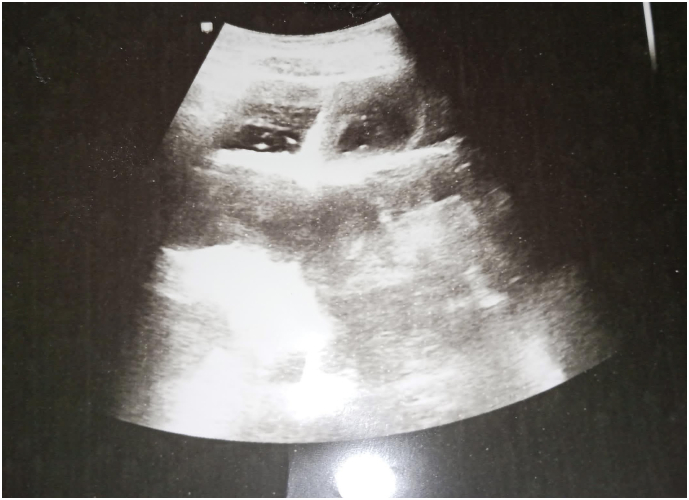
Abdominal ultrasound shows dilated small bowel with retrograde movements and a collapsed segment in the right iliac region. The liver, gallbladder, biliary tract, pancreas, spleen, and kidneys appear normal.

**Fig. 2:**
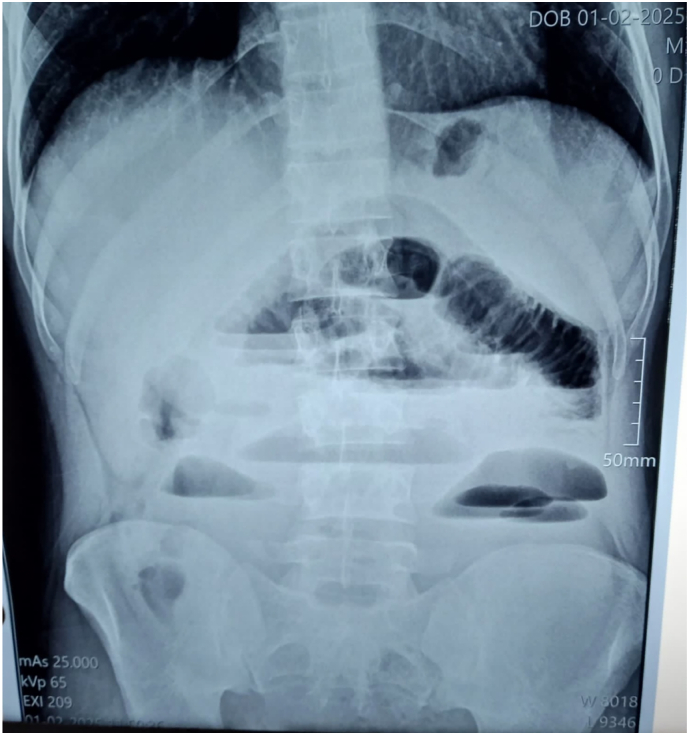
The abdominal X-ray image in the standing position shows the presence of gas-fluid levels with a complete absence of gas in the pelvis. There is no gas crescent under the diaphragm.

**Fig. 3:**
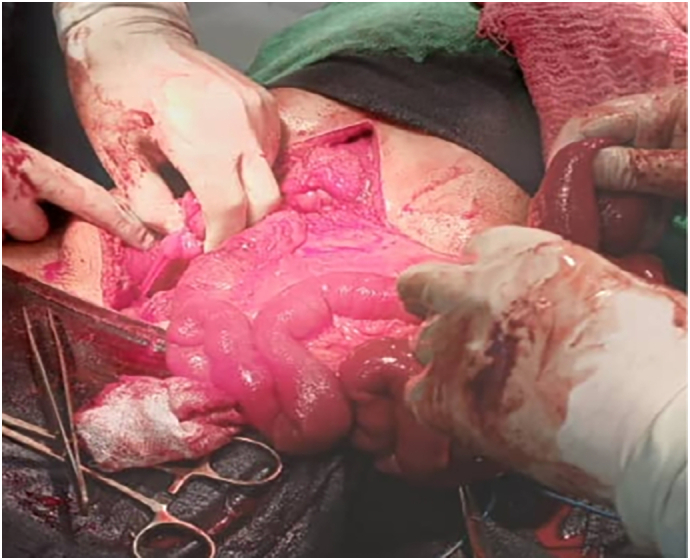
The image after bowel reduction, hernia repair, and resection of the redundant sigmoid colon.

**Fig. 4:**
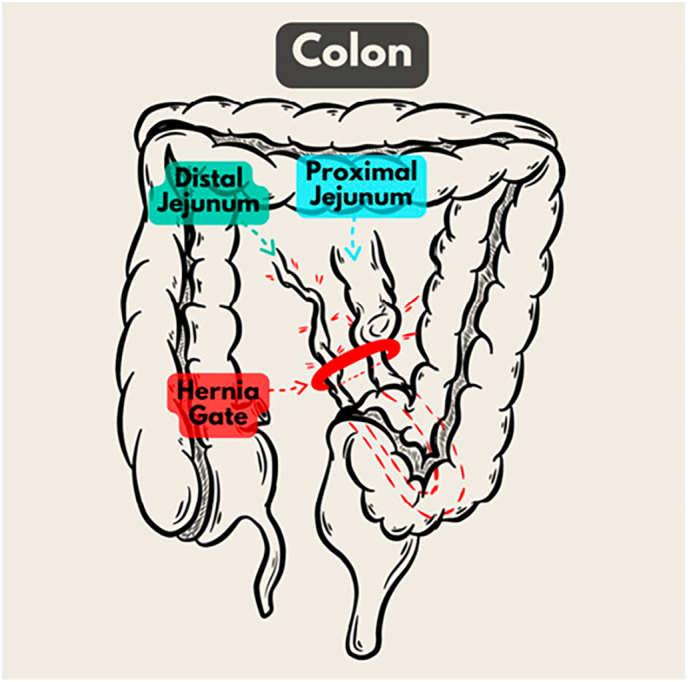
A schematic illustration showing a transmesosigmoid hernia, characterized by a defect in the sigmoid mesocolon, through which the small intestine has herniated.

A 30-year-old patient presented with a several-hour history of sudden, severe abdominal pain and intractable vomiting, with no reported flatulence or constipation. The patient reported no prior medical conditions or previous surgeries. Vital signs were stable. A blood test demonstrated a high white blood cell count of 17,000/dL. On abdominal examination, generalized defence was noted, with maximal tenderness in the hypogastric region. Hyperactive bowel sounds were also audible. An abdominal ultrasound revealed diffuse dilation of bowel loops with antiperistaltic movements and a collapsed segment in the right abdomen (Fig. 1). An upright plain abdominal X-ray demonstrated air-fluid levels with complete absence of pelvic gas (Fig. 2). We decided to perform an emergency laparotomy. Intraoperatively, a large IH was found within the root of the elongated sigmoid mesocolon, extending through its right leaflet. This hernia contained the entire small bowel, severely displacing the left leaflet, which in turn formed a large peritoneal sac filling the abdomen and enclosing the small bowel, along with the displacement of the elongated sigmoid colon and descending colon to the right (Fig. 4 [8]). The herniated bowel was reduced, and we noted it to be well perfused with no evidence of necrosis (Fig. 3). We performed a resection of the elongated sigmoid colon and brought out the descending colon as a temporary colostomy, burying the distal end as a Hartmann's pouch. In our case, there was evident fecal contamination within the abdominal cavity. Given the emergent nature of the surgery, there was no opportunity to perform mechanical bowel preparation. Although the herniated small bowel appeared well-perfused, we observed an abnormally elongated sigmoid colon with significant displacement, resulting in mesenteric twisting and dilatation. This anatomical distortion raised concerns about the potential for recurrent herniation or future sigmoid volvulus. Consequently, we opted to resect the affected segment and create a temporary colostomy. Furthermore, the presence of a large peritoneal sac containing the entire small bowel raised additional concern regarding the safety and integrity of a primary anastomosis. Under these circumstances, a diverting colostomy was deemed the safer and more appropriate option. Postoperatively, the patient was kept nil per os and started on intravenous fluids and antibiotic coverage. Early ambulation was encouraged. A clear liquid diet was initiated upon the return of flatus output via the colostomy, and a full liquid diet was permitted 48 h after the onset of colostomy output. The patient was discharged on postoperative day 8, with normal laboratory findings and recommendations for a high-protein diet. No significant complications developed. A minor wound dehiscence occurred, which was managed with local wound care and subsequently healed. The patient was seen in the clinic two weeks later for suture removal and was in good general condition without any further complications.

## 3 Discussion

Abdominal hernias involving the bowel are generally classified into two main types: internal hernias and external hernias. This case aims to investigate the less common IH, which is a viscus protrusion via an abdominal cavity fossa or foramen [9]. His account for 0.5 to 4.1 % of all cases and frequently manifest as small intestinal obstruction [10]. Depending on where they occur, His can be further categorized. In 1964, Benson and Killen [18] divided sigmoid mesocolon hernias into three types: (1) intersigmoid hernia, which develops when the left peritoneal surface of the sigmoid mesentery and the parietal peritoneum of the posterior abdominal wall adhere; (2) transmesosigmoid hernia, which happens when the intestinal loops get stuck due to a complete thickness sigmoid mesocolon defect; and (3) intramesosigmoid hernia. This condition affects the sigmoid mesocolon and typically affects one side—most often the lateral side. It results from a congenital, oval-shaped defect located near the colon, independent of the intersigmoid fossa, leading to herniation. In the present case, the type of hernia is a TMSH. Though both acquired and congenital etiologies have been suggested, the precise etiology of mesentery abnormalities is still unknown. Trauma, prior laparoscopic or laparotomy surgery, and intra-abdominal inflammation are examples of acquired risk factors [11]. Acute abdominal pain and symptoms of small intestinal blockage are common in patients with TMSHs [12,13]. Intestinal strangling from His increases the risk of intestinal gangrene and perforation [9,14]. As for the diagnosis, CT is the gold standard imaging technique, as it is rapid, readily available, and provides much detail. The visualization of normal anatomy and pathology has improved with the introduction of multidetector CT (MDCT), which generates high-quality multiplanar reconstructions (MPRs) and thin-section axial images. As a result, His are now more frequently diagnosed as the cause of SBO prior to surgery [15]. In order to treat intramesosigmoid hernias, the hernia must be reduced and the defect repaired; this can be done laparoscopically or openly. While open approaches provide good ergonomics for the surgeons and equipment mobility, laparoscopic procedures have the advantages of fewer complications and a shorter hospital stay [16,17]. Hartmann's method, which entails resecting the afflicted segment and establishing a temporary end colostomy without anastomosis, and primary anastomosis, in which the bowel ends are reconnected directly, are the two procedures that are frequently debated in cases of intestinal necrosis. Because Hartmann's treatment lowers the risk of anastomotic leakage and related mortality, it is typically used in patients who are hemodynamically unstable or in cases of severe peritoneal contamination [19]. Nonetheless, some research indicates that, in stable patients who have received proper preoperative optimization, primary anastomosis may be safe and prevent the need for a second procedure to reverse the colostomy [20]. According to several studies, primary anastomosis provides better long-term results when patient stability and operating conditions permit it, even if Hartmann's approach lowers early postoperative morbidity in critically ill patients [21,22].

## 4 Conclusion

TMSHs are a rare and often overlooked cause of SBO. Their nonspecific clinical presentation makes diagnosis challenging, frequently leading to delays that can increase the risk of serious complications such as strangulation and bowel necrosis. Surgical repair remains the definitive treatment, with laparoscopic approaches offering benefits in recovery. This case highlights the significance of this rare type of hernia and the importance of early diagnosis and surgical intervention.

## List of abreviations

IHs: Internal hernias
SBO: Small bowel obstruction
TMSHs: Transmesosigmoid hernias
CT: Computed tomography
MDCT: Multidetector CT
MPRs: Multiplanar reconstructions

## CrediT authorship contribution statement

Ibrahim Fathallah: Writing – review & editing, Writing – original draft, Data curation.

Ahmed Al-Talep: Writing – review & editing, Writing – original draft.

Abd Alrhman Alajrd: Writing – review & editing, Writing – original draft.

Abd Al-Hadi Al-Souqi: Writing – review & editing, Writing – original draft.

Saleh Saleh: Writing – review & editing, Supervisor.

Ibrahim Fathallah: submitted the final manuscript.

All authors read and approved the final manuscript.

## Consent for publication

Written informed consent was obtained from the patient's parents/legal guardian for publication and any accompanying images. A copy of the written consent is available for review by the Editor-in-Chief of this journal on request.

## Ethics approval and consent to participate

Ethics clearance was not necessary since the University waives ethics approval for publication of case reports involving no patients' images, and the case report is not containing any personal information. The ethical approval is obligatory for research that involve human or animal experiments.

## Funding

The author(s) received no financial support for the research, authorship, and/or publication of this article.

## Declaration of competing interest

The author(s) declared no potential conflicts of interest with respect to the research, authorship, and/or publication of this article.

## Data Availability

Data sharing not applicable to this article as no datasets were generated or analyzed during the current study.
